# A Novel Role for Ecdysone in *Drosophila* Conditioned Behavior: Linking GPCR-Mediated Non-canonical Steroid Action to cAMP Signaling in the Adult Brain

**DOI:** 10.1371/journal.pgen.1003843

**Published:** 2013-10-10

**Authors:** Hiroshi Ishimoto, Zhe Wang, Yi Rao, Chun-Fang Wu, Toshihiro Kitamoto

**Affiliations:** 1 Department of Anesthesia and Pharmacology, Carver College of Medicine, University of Iowa, Iowa City, Iowa, United States of America; 2 Department of Biology, College of Liberal Arts and Sciences, University of Iowa, Iowa City, Iowa, United States of America; 3 National Institute of Biological Sciences, Beijing, People's Republic of China; 4 Peking-Tsinghua Center for Life Sciences, Peking University School of Life Sciences, Beijing, People's Republic of China; 5 Interdisciplinary Programs in Genetics and Neuroscience, University of Iowa, Iowa City, Iowa, United States of America; Janelia Farm Research Campus, Howard Hughes Medical Institute, United States of America

## Abstract

The biological actions of steroid hormones are mediated primarily by their cognate nuclear receptors, which serve as steroid-dependent transcription factors. However, steroids can also execute their functions by modulating intracellular signaling cascades rapidly and independently of transcriptional regulation. Despite the potential significance of such “non-genomic” steroid actions, their biological roles and the underlying molecular mechanisms are not well understood, particularly with regard to their effects on behavioral regulation. The major steroid hormone in the fruit fly *Drosophila* is 20-hydroxy-ecdysone (20E), which plays a variety of pivotal roles during development via the nuclear ecdysone receptors. Here we report that DopEcR, a G-protein coupled receptor for ecdysteroids, is involved in activity- and experience-dependent plasticity of the adult central nervous system. Remarkably, a courtship memory defect in *rutabaga* (Ca^2+^/calmodulin-responsive adenylate cyclase) mutants was rescued by *DopEcR* overexpression or acute 20E feeding, whereas a memory defect in *dunce* (cAMP-specific phosphodiestrase) mutants was counteracted when a loss-of-function *DopEcR* mutation was introduced. A memory defect caused by suppressing dopamine synthesis was also restored through enhanced DopEcR-mediated ecdysone signaling, and rescue and phenocopy experiments revealed that the mushroom body (MB)—a brain region central to learning and memory in *Drosophila*—is critical for the DopEcR-dependent processing of courtship memory. Consistent with this finding, acute 20E feeding induced a rapid, DopEcR-dependent increase in cAMP levels in the MB. Our multidisciplinary approach demonstrates that DopEcR mediates the non-canonical actions of 20E and rapidly modulates adult conditioned behavior through cAMP signaling, which is universally important for neural plasticity. This study provides novel insights into non-genomic actions of steroids, and opens a new avenue for genetic investigation into an underappreciated mechanism critical to behavioral control by steroids.

## Author Summary

The brain is a prominent target of steroid hormones, which control a variety of neurobiological processes and are critical to the regulation of behavior. Some effects of these hormones involve changes in gene expression and thus emerge slowly, over the course of hours or even days. Other responses to steroids occur rapidly and are independent of transcriptional regulation. Their functions and mechanisms of action are poorly understood, particularly in the context of steroid-mediated control of behavior. Here we show, using the genetic model organism *Drosophila melanogaster* (the fruit fly), that an unconventional, membrane-bound receptor for the molting hormone ecdysone transmits a novel form of steroid signaling in the adult brain. Our study shows that this novel form of steroid signaling has a robust interface with the classical “memory genes” that encode central components of the so-called cAMP signaling pathway, which is universally important for neuronal and behavioral plasticity. These findings underscore the significance of steroid signaling in memory processing, and provide a foundation for the genetic analysis of rapid, unconventional steroid signaling in behavioral regulation.

## Introduction

Steroid hormones are essential modulators of a broad range of biological processes in a diversity of organisms across phyla. In the adult nervous system, the functions of steroids such as estrogens and glucocorticoids are of particular interest because they have significant effects on the resilience and adaptability of the brain, playing essential roles in endocrine regulation of behavior. Reflecting their importance in neural functions, steroid hormones are implicated in the etiology and pathophysiology of various neurological and psychiatric disorders, and are thus often targeted in therapies [1]–[7]. The biological actions of steroids are mediated mainly by nuclear hormone receptors—a unique class of transcription factors that activate or repress target genes in a steroid-dependent manner [8]. Substantial evidence suggests, however, that steroid hormones can also exert biological effects quickly and independently of transcriptional regulation, by modulating intracellular signaling pathways [9]. Such “non-genomic” effects might be induced by direct allosteric regulation of ion channels, including receptors for GABA [10] and NMDA [11]. Alternatively, in certain contexts, non-genomic steroid signaling could be mediated by classical nuclear hormone receptors acting as effector molecules in the cytosol [12], [13].

G-protein coupled receptors (GPCRs) that directly interact with steroids have the potential to play an important role in non-genomic steroid signaling. So far, however, only few GPCRs have been identified as *bona fide* steroid receptors in vertebrates [14], [15]. The G-protein coupled estrogen receptor 1 (GPER, formally known as GPR30) is the best studied GPCR that is responsive to steroids. Pharmacological and gene knockout approaches suggest that this protein has widespread roles in the reproductive, nervous, endocrine, immune and cardiovascular systems [15]. Although other G-protein coupled receptors were predicted to be responsive to steroids (e.g., the Gq-coupled membrane estrogen receptor and estrogen receptor-X), their molecular identity is not known [16], [17]. Overall, the physiological roles of the GPCR-mediated actions of steroids and the underlying molecular mechanisms remain poorly understood, and sometimes controversial, in spite of their importance [18], [19]. In particular, it is unknown how this non-canonical steroid mechanism influences neural functions and complex behaviors.

*Drosophila* genetics has been extensively used to study the roles and mechanisms of action of steroid hormones *in vivo*. The major steroid hormone in *Drosophila* is the molting hormone 20-hydroxy-ecdysone (20E), which orchestrates a wide array of developmental events, including embryogenesis, larval molting and metamorphosis [20]–[22]. Recent studies revealed that 20E also plays important roles in adult flies, regulating: the innate immune response [23], stress resistance, longevity [24], the formation of long-term courtship memory [25] and the active/resting state [26]. In general, the functions of 20E during development and adulthood are thought to be executed by ecdysone receptors (EcRs), members of the evolutionarily conserved nuclear hormone receptor family [21], [27], [28].

In addition to canonical ecdysone signaling via EcRs, Srivastava et al. identified a novel GPCR called DopEcR, and showed that it propagates non-genomic ecdysone signaling *in vitro* [29]. DopEcR shares a high level of amino-acid sequence similarity with vertebrate β-adrenergic receptors. *In situ* hybridization [29] and microarray data (FlyAtlas, http://flyatlas.org/) revealed that *DopEcR* transcripts are preferentially expressed in the nervous system. In heterologous cell culture systems, DopEcR is localized to the plasma membrane and responds to dopamine as well as ecdysteroids (ecdysone and 20E), modulating multiple, intracellular signaling cascades [29]. Furthermore, Inagaki et al. recently detected DopEcR expression in the sugar-sensing gustatory neurons of adult flies, and showed that DopEcR-mediated dopaminergic signaling enhances the proboscis extension reflex during starvation [30]. Nonetheless, little is known about whether DopEcR functions as a steroid receptor *in vivo*, and about how it drives responses in the central nervous system (CNS) to modulate complex behaviors. Here, we report for the first time that DopEcR mediates non-genomic ecdysone signaling in the adult brain, and that it is critical for memory processing. We also show that, during memory processing, DopEcR transmits information via novel steroid signals that interact with the cAMP pathway, a signaling cascade that is universally important for neuronal and behavioral plasticity. Our genetic study thus uncovers underappreciated GPCR-mediated functions and mechanisms of action that employ non-canonical steroid signaling to regulate the adult nervous system and, thereby, behavior.

## Results

### An intronic *piggyBac* insertion results in a hypomorphic *DopEcR* mutant allele

**Figure 1:**
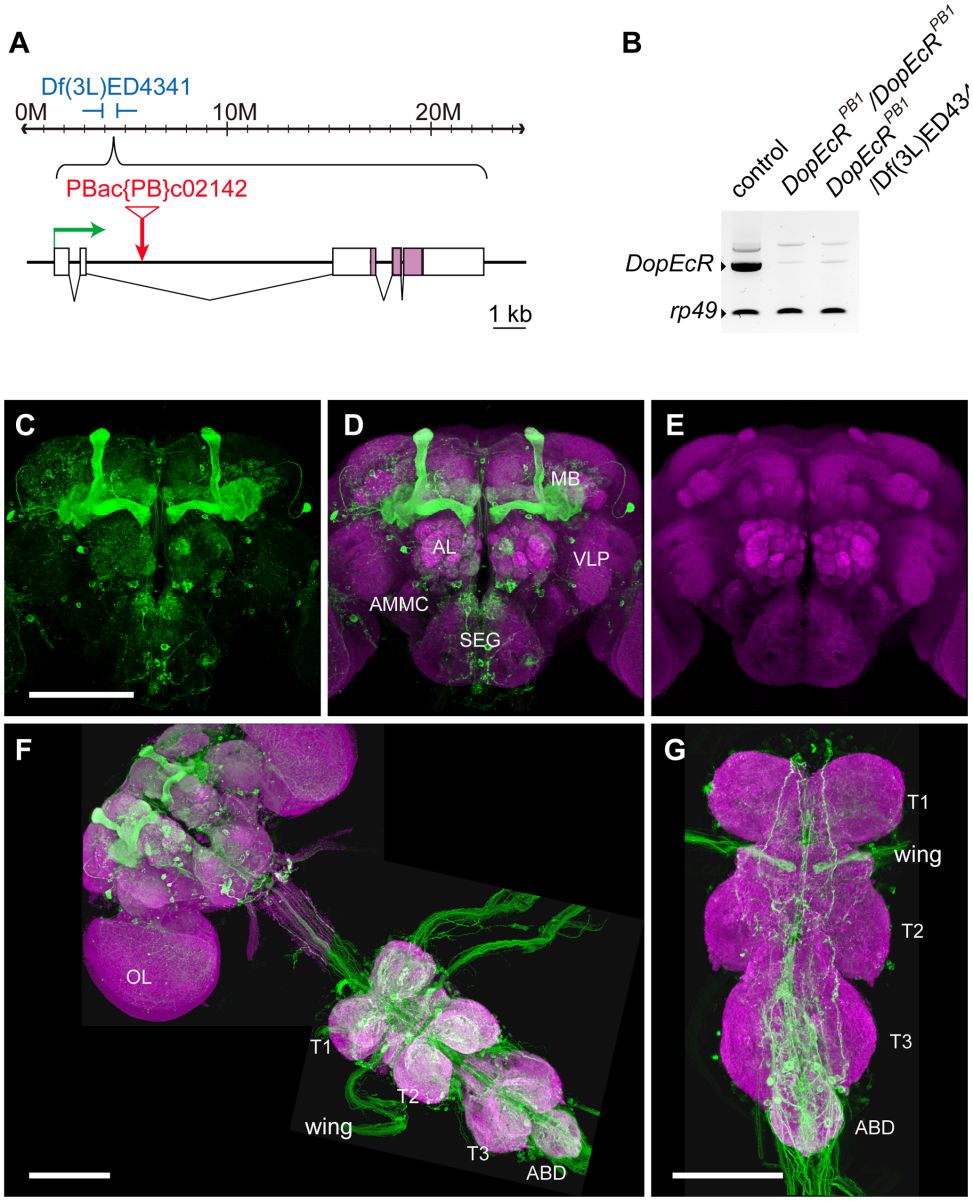
A *DopEcR* hypomorphic allele and the *DopEcR*-Gal4 expression pattern. (A) Schematic representation of the *Drosophila DopEcR* gene. *DopEcR* contains 5 exons and spans a 12.7 kb genomic region in the 64B2-64B3 cytological interval (Flybase). The *DopEcR* exons are represented by boxes, and the coding regions are indicated with purple color. A green arrow indicates the direction of transcription of *DopEcR*. The *DopEcR* locus is completely uncovered by deficiency Df(3L)ED4341. The PBac(PB)c02142 (*DopEcR^PB1^*) allele harbors an insertion of the *piggyBac* transposable element within the second intron. The insertion site is indicated as a red arrow with an inverted triangle. (B) *DopEcR* transcript levels in *DopEcR^PB1^* homozygotes and trans-heterozygotes, as assessed by RT-PCR analysis. (C–G) Expression of the GFP reporter gene (UAS-CD4-tdGFP [70]) driven by *DopEcR*-Gal4 (green) and synaptic neuropil labeled with the nc82 antibody against presynaptic marker protein Bruchpilot (magenta). Anterior view of the adult brain displaying *DopEcR*-Gal4 expression pattern (C) and the nc82 staining (E). A merged image is shown in (D). (F) Adult brain with the thoracicoabdominal ganglion (ventral view). (G) Dorsal view of the thoracicoabdominal ganglion. Scale bars represent 100 µm. ABD: abdominal ganglion; AL: antennal lobe; AMMC: antennal mechanosensory and motor center; MB: mushroom body; OL: optic lobe; SEG: subesophageal ganglion; T1: prothoracic ganglion; T2: metathoracic ganglion; T3: mesothoracic ganglion; VLP: ventrolateral protocerebrum; wing: wing neuropil.

PBac(PB)c02142 is a *piggyBac* transposon insertion in the second intron of the *DopEcR* gene (Figure 1A). Adult flies homozygous for PBac(PB)c02142 displayed a significant reduction in *DopEcR* transcript levels (<20% of levels in control), in both the head (Figure 1B) and the body (data not shown). Df(3L)ED4341 is a chromosomal deficiency that removes multiple genes on 3L, including *DopEcR* (Flybase: http://flybase.org/). Flies trans-heterozygous for PBac(PB)c02142 and Df(3L)ED4341 showed levels of *DopEcR* transcript comparable to those in PBac(PB)c02142 homozygotes (Figure 1B). PBac(PB)c02142 is therefore a hypomorphic allele of *DopEcR*, and it was mainly used in this study to investigate the functions of *DopEcR* in behavioral plasticity. PBac(PB)c02142 is referred to as *DopEcR^PB1^* hereafter. *DopEcR^PB1^* homozygotes reached adulthood and exhibited no gross morphological defects. General motor activity was not significantly impaired, as judged by analysis of reactive climbing behavior (Figure S1).

In order to obtain some insight into the endogenous expression pattern of *DopEcR*, we generated *DopEcR*-Gal4, a Gal4 driver that contains the putative enhancer/promoter sequence of *DopEcR* (a 588-bp DNA fragment upstream of the *DopEcR* transcription start site). *DopEcR*-Gal4 was found to induce GFP reporter gene expression preferentially in the nervous system. In the adult brain, *DopEcR*-Gal4-regulated reporter gene expression was particularly prominent in the mushroom body (MB) (Figure 1C and 1D). It is not likely that the endogenous *DopEcR* expression is accurately recapitulated by the 588-bp DNA fragment used for *DopEcR*-Gal4. Nevertheless, the reporter gene expression shown in Figure 1C and 1D implies the presence of the endogenous DopEcR in the MBs of the adult brain (see Discussion). Reporter gene expression driven by *DopEcR*-Gal4 was also observed in neuronal soma and fibers localized in each segment of the thoracicoabdominal ganglion (Figure 1F and 1G). In addition, a number of fibers connecting the ganglion to the brain, abdomen and appendages were found to be GFP-positive (Figure 1F and 1G).

### *DopEcR* mutants exhibit a reduced rate of giant-fiber habituation

**Figure 2:**
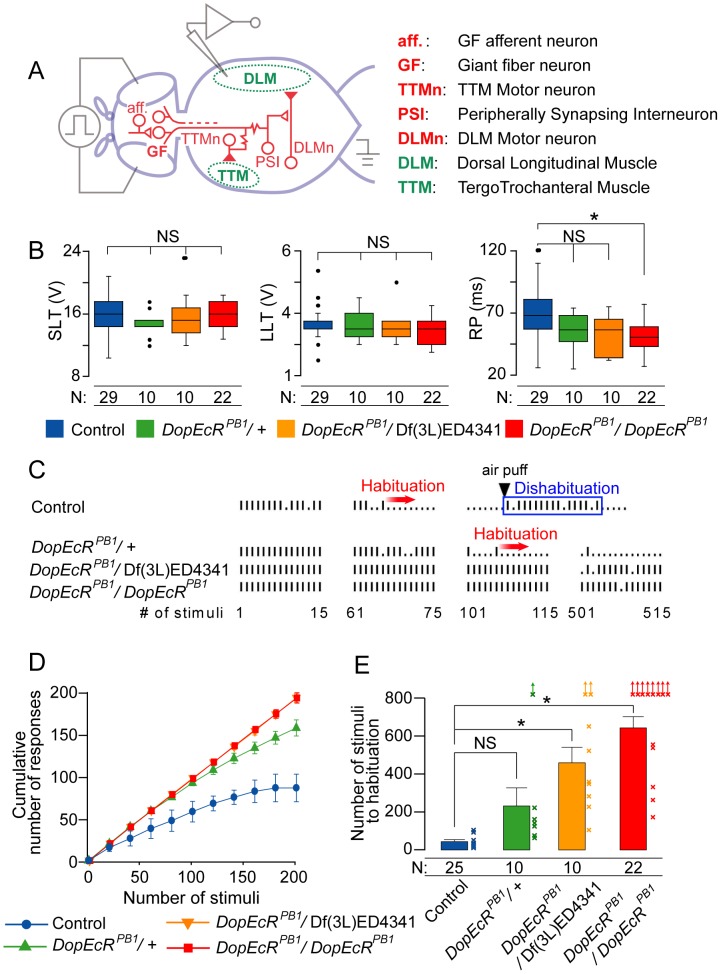
*DopEcR* loss-of-function mutants show slow habituation of the giant-fiber escape circuit. (A) Schematic representation of the giant-fiber (GF) pathway. GF afferent neurons (aff.). Tergotrochanteral muscle (TTM), TTM motor neuron (TTMn), dorsal longitudinal muscle (DLM), peripherally synapsing interneuron (PSI), and DLM motor neuron (DLMn). (B) The long-latency threshold (LLT), short-latency threshold (SLT) and refractory period (RP) for *DopEcR* mutants and wild-type flies. The data are presented as box plots. (C) Representative traces of muscle responses to 5-Hz brain stimulation. Vertical bars and dots denote successful and failed responses, respectively. An arrowhead represents the time at which a gentle air puff was applied to trigger dishabituation, which confirms that the failure to respond is due to habituation. (D) Cumulative muscle responses to 5-Hz brain stimulations. (E) The average numbers of 5-Hz stimuli delivered before the fly experiences five consecutive failures (criteria for habituation). Crosses with arrows represent flies that did not show habituation within the observation period (2 minutes). Error bars (s.e.m). Data were analyzed by Krustal-Wallis One-Way ANOVA, followed by Dunn's pairwise test for multiple comparisons. NS, no significant difference. *, *P*<0.05.

To investigate the role of DopEcR in the CNS, we tested *DopEcR* mutations for effects on the electrophysiological properties of the adult giant-fiber (GF) pathway [31], [32]. Visual or mechanical stimulation activates the descending GF neurons (Figure 2A), triggering the stereotypical jump-and-flight response. This behavioral response is associated with a consistent pattern of spiking in both the dorsal longitudinal flight muscle (DLM) and the tergotrochanteral jump muscle (TTM) (Figure 2A). Strong electrical stimulation of the brain can bypass sensory receptors and directly trigger the neuronal circuit at the GF neurons (short-latency response) [32], [33]. Alternatively, with stimulation of the brain at lower intensity, the circuit is activated at GF afferents in the brain (long-latency response) [34]. As shown in Figure 2B (left and middle panels), both the short- and long-latency thresholds (SLT and LLT; the lowest intensities required to trigger short- and long-latency responses in the DLM) were indistinguishable between *DopEcR* mutants (*DopEcR^PB1^*/*DopEcR^PB1^*, *DopEcR^PB1^*/Df(3L)ED4341 and *DopEcR^PB1^*/+) and wild-type flies. This indicates that reducing *DopEcR* expression does not significantly affect the overall neuronal sensitivity of the GF pathway. In contrast, the refractory period (RP; the minimum time required for the GF system to recover from the 1^st^ stimulus and fire a response to the 2^nd^ stimulus) was significantly reduced in *DopEcR^PB1^*/*DopEcR^PB1^* compared to control flies (Figure 2B, right panel). The RP in *DopEcR^PB1^*/Df(3L)ED4341 and *DopEcR^PB1^*/+ flies also showed a similar tendency, although the differences between these mutants and control flies did not reach statistical significance, possibly due to the weak nature of this *DopEcR^PB1^* phenotype and the small sample numbers. Nonetheless, the shorter RP implies that circuits in *DopEcR* mutants are less vulnerable or more resistant to activity-dependent modifications than the relevant circuits in controls are.

Diminished neuronal plasticity in *DopEcR* mutants was unequivocally demonstrated when habituation of the GF pathway was analyzed. Habituation is a simple form of non-associative learning, in which the reaction to a particular stimulus becomes diminished when the stimulus is applied repeatedly. Habituation does not lessen behavioral responses due to sensory adaptation or motor fatigue [35]. When electrical stimulation is repeatedly delivered across the brain, the GF pathway undergoes habituation and the probability of a motor output significantly decreases [36]. Previous studies by us and others revealed that the loci responsible for this neuronal plasticity are localized to the brain, namely neuronal circuits afferent to the GF neurons (aff; Figure 2A) [36]–[40]. Other elements in the GF pathway—including the GF neuron, the peripherally synapsing interneuron (PSI), and the motor neurons that innervate the flight and jump muscles (DLMs and TTMs; Figure 2A)—are robust enough to reliably respond to sustained high-frequency stimuli (up to ∼100-Hz) [32], [33], [36]. In our experiments, control flies became rapidly habituated to 5-Hz stimulation of the brain, as evidenced by a failure of their DLM to respond (Figure 2C, control). The reduced behavioral response was not a consequence of sensory adaptation or motor fatigue because the response was readily recovered by a novel stimulus, such as an air puff (dishabituation; Figure 2C, control). In contrast to controls, *DopEcR^PB1^* homozygotes and *DopEcR^PB1^*/Df(3L)ED4341 trans-heterozygotes consistently showed a delay in habituation (Figure 2C), and thus their cumulative response was greater than that of controls (Figure 2D). *DopEcR^PB1^* heterozygotes (*DopEcR^PB1^*/+) showed a similar tendency, although the effect was less extreme (Figure 2C and 2D). When habituation was arbitrarily defined as five or more consecutive failures, *DopEcR^PB1^* mutants needed more repetitive stimulations than control flies to reach habituation status (Figure 2E). The average numbers of 5-Hz stimuli required for habituation were 46±31 and 637±236 in control flies and *DopEcR^PB1^* homozygotes, respectively (Figure 2E). *DopEcR^PB1^* heterozygotes also showed a slow habituation phenotype (Figure 2C–E). These results demonstrated that DopEcR is an essential modulatory component of the GF pathway, and that its endogenous role is to positively regulate activity-dependent modification of the relevant CNS neuronal circuits.

### *DopEcR* is required in adults for normal courtship memory

**Figure 3:**
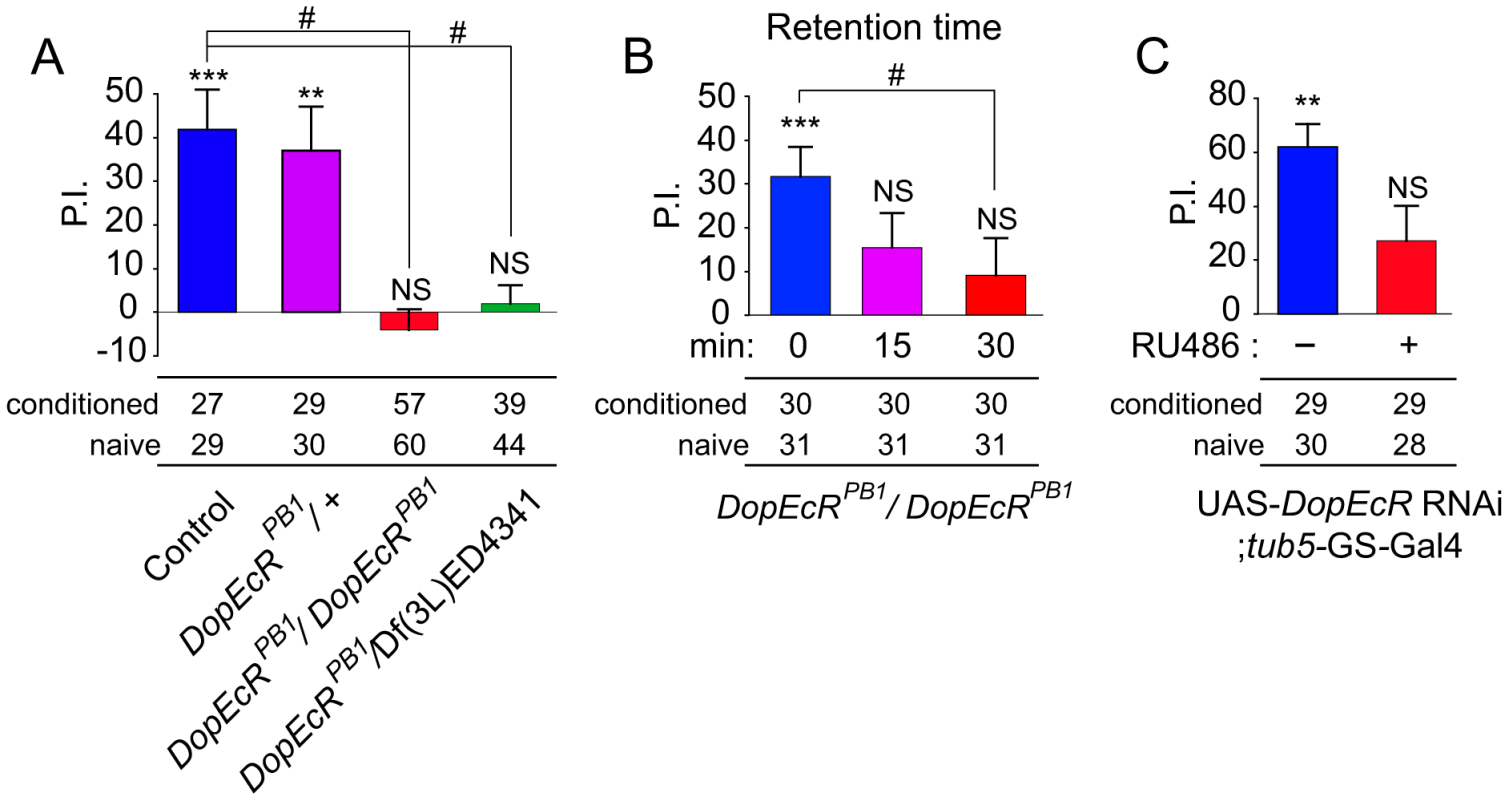
DopEcR is required for the 30-minute courtship memory induced by 1-hour courtship conditioning. (A) Thirty-minute courtship memory in wild-type flies (control) and flies heterozygous (*DopEcR^PB1^*/+), homozygous (*DopEcR^PB1^*/*DopEcR^PB1^*), and hemizygous (*DopEcR^PB1^*/Df(3L)ED4341) for *DopEcR*. *DopEcR^PB1^* homozygotes and hemizygotes were defective for 30-minute courtship memory. (B) Time course of courtship memory in *DopEcR^PB1^* homozygotes. Significant memory was observed immediately after conditioning, but not 15 or 30 minutes after conditioning. (C) A defect in 30-minute courtship memory in flies that ubiquitously express the *DopEcR* RNAi after eclosion, in response to RU486 stimulation of the *tub*-GS-Gal4 driver. The presence or absence of courtship memory was evaluated by applying the Mann–Whitney U-test to naïve and conditioned males. Statistical significance is shown above each bar as NS, no significant difference, **, *P*<0.01 or ***, *P*<0.001. Sample numbers for naïve and conditioned flies are shown under each graph. PIs were analyzed using Krustal-Wallis One-Way ANOVA, followed by Dunn's pairwise test for multiple comparisons. #, *P*<0.05; ##, *P*<0.01. Error bars (s.e.m.).

In light of the abnormalities in GF habituation, we next tested *DopEcR* mutants for experience-dependent courtship suppression, an ethologically relevant associative-learning paradigm [41], [42]. In wild-type control males (+/+) and *DopEcR^PB1^* heterozygous males (*DopEcR^PB1^*/+), 1 hour of conditioning with a mated female induced “courtship memory”, which was readily detectable 30 minutes after conditioning as a statistically significant, experience-dependent reduction in courtship activity (*P* = 0.0004 for control and 0.0046 for *DopEcR^PB1^*/+; Figure 3A). In contrast, *DopEcR^PB1^* homozygotes and hemizygotes (*DopEcR^PB1^*/Df(3L)ED4341) did not display courtship memory (*P*>0.05; Figure 3A). These results strongly suggested that DopEcR is essential to the processing of courtship memory. The performance indices (PIs; % decrease in courtship index in response to courtship conditioning, see Materials and Methods for details) of these *DopEcR^PB1^* mutants at 30 minutes post conditioning were significantly lower than that of wild-type flies (*P*<0.05; Figure 3A). Notably, although *DopEcR^PB1^* homozygotes did not display courtship memory at both 15 and 30 minutes after conditioning (*P*>0.05), they exhibited memory immediately after courtship conditioning (*P* = 0.00026). The PIs of *DopEcR^PB1^* homozygotes for 0 and 30 minutes after conditioning were significantly different from each other (Krustal-Wallis One-Way ANOVA; *P*<0.05; Figure 3B). These results indicated that *DopEcR* mutants retain the ability to acquire courtship memory, but that the memory is labile and severely disrupted within 30 minutes.

To confirm that the memory phenotype in *DopEcR* mutants is due to the defect in *DopEcR* function, we examined the effects of *DopEcR* RNAi on courtship memory. When the *DopEcR* RNAi was conditionally and globally expressed in adult flies using the RU486-inducible driver *tubulin5*-GeneSwitch-Gal4 (*tub5*-GS-Gal4; a gift from Dr. Pletcher, University of Michigan) [43], the level of *DopEcR* transcripts was significantly reduced in an RU486-dependent manner (Figure S2). When *DopEcR* expression was conditionally knocked down by RNAi in this context, the courtship memory phenotype of the *DopEcR* mutants was mimicked (Figure 3C). These results support our conclusion that adult male flies require functional DopEcR for normal courtship memory.

### *DopEcR* expression in neurons of the mushroom body is required for courtship memory

**Figure 4:**
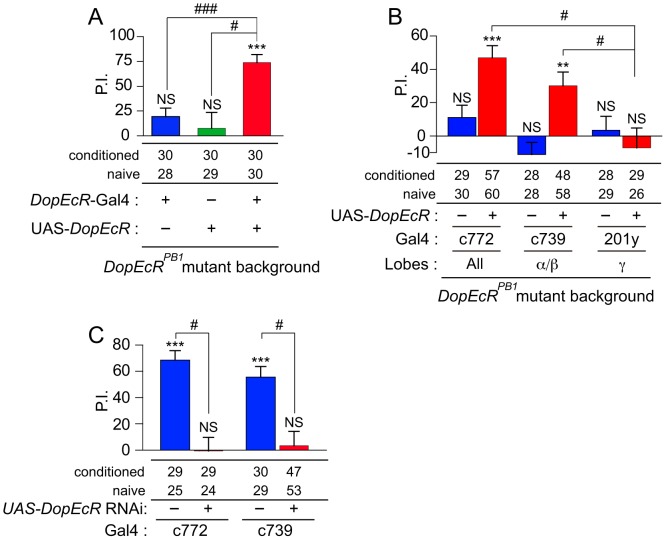
The mushroom body is critical for the DopEcR-dependent processing of courtship memory. (A) Rescue of the *DopEcR^PB1^* memory defect by expression of wild-type *DopEcR* transgene using the *DopEcR*-Gal4 driver. (B) Rescue of *DopEcR^PB1^* memory defect by expression of wild-type *DopEcR* transgene under control of the MB-specific c772, c739 and 201 y drivers. Note that MB-Gal4 lines drive reporter expression in different subsets of MB neurons (see text). (C) Courtship memory defect induced by MB-specific expression of the *DopEcR* RNAi using the c772 and c739 drivers. The presence or absence of courtship memory was evaluated by applying Mann–Whitney U-test to naïve and conditioned flies. Statistical significance is shown above each bar. NS, no significant difference. **, *P*<0.01; ***, *P*<0.001. Sample numbers for naïve and conditioned flies are shown under each graph. PIs were analyzed using Student's t-test or Krustal-Wallis One-Way ANOVA, followed by Dunn's pairwise test for multiple comparisons. #, *P*<0.05; ###, *P*<0.001. Error bars (s.e.m.).

Next we sought to identify the sites within the nervous system in which DopEcR is required for the processing of courtship memory. We found that *DopEcR^PB1^* males displayed courtship memory (*P* = 9.6×10^−6^) when the wild-type *DopEcR* transgene was expressed using *DopEcR*-Gal4 (Figure 4A). In contrast, control *DopEcR^PB1^* males carrying only *DopEcR*-Gal4 or UAS-*DopEcR* were defective for courtship memory (Figure 4A). The PIs of these control males were significantly lower than that of *DopEcR^PB1^* males carrying both the Gal4 and UAS constructs (*P*<0.001 and *P*<0.05, respectively; Figure 4A). *DopEcR*-Gal4 directed gene expression in the adult brain, particularly in the neurons of the MB (Figure 1C). These observations, together with the importance of the MBs in processing courtship memory [25], [44], [45], led us to suspect that the rescue of the *DopEcR* memory phenotype by *DopEcR*-Gal4 was a consequence of the expression of wild-type DopEcR in the MB. This possibility was tested by performing rescue experiments for *DopEcR^PB1^* mutants in which UAS-*DopEcR* expression was driven using three MB-positive Gal4 lines: c772, c739 and 201Y. Courtship memory was restored in *DopEcR^PB1^* males when the wild-type *DopEcR* cDNA was expressed using either c772 or c739 (*P* = 2.9×10^−5^ or 0.0063; Figure 4B). In contrast, the 201 y driver failed to rescue the memory defect of *DopEcR^PB1^* mutants (Figure 4B). c772 and c739 drive gene expression in all three types of MB neurons (α/β, α′/β′ and γ) and primarily in the α/β neurons, respectively, whereas 201 y drives gene expression mainly in the γ neurons [46]. These results suggested that the MBs, in particular the α/β neurons, are the key anatomical site in which DopEcR regulates courtship memory. In support of this idea, expression of the *DopEcR* RNAi in wild-type MB neurons using c772 or c739 led to a lack of 30-minute courtship memory in males (Figure 4C). The PIs of males carrying both the Gal4 and UAS-RNAi constructs were significantly lower than that of control males (Figure 4C).

### Impaired ecdysone synthesis leads to defective courtship memory

**Figure 5:**
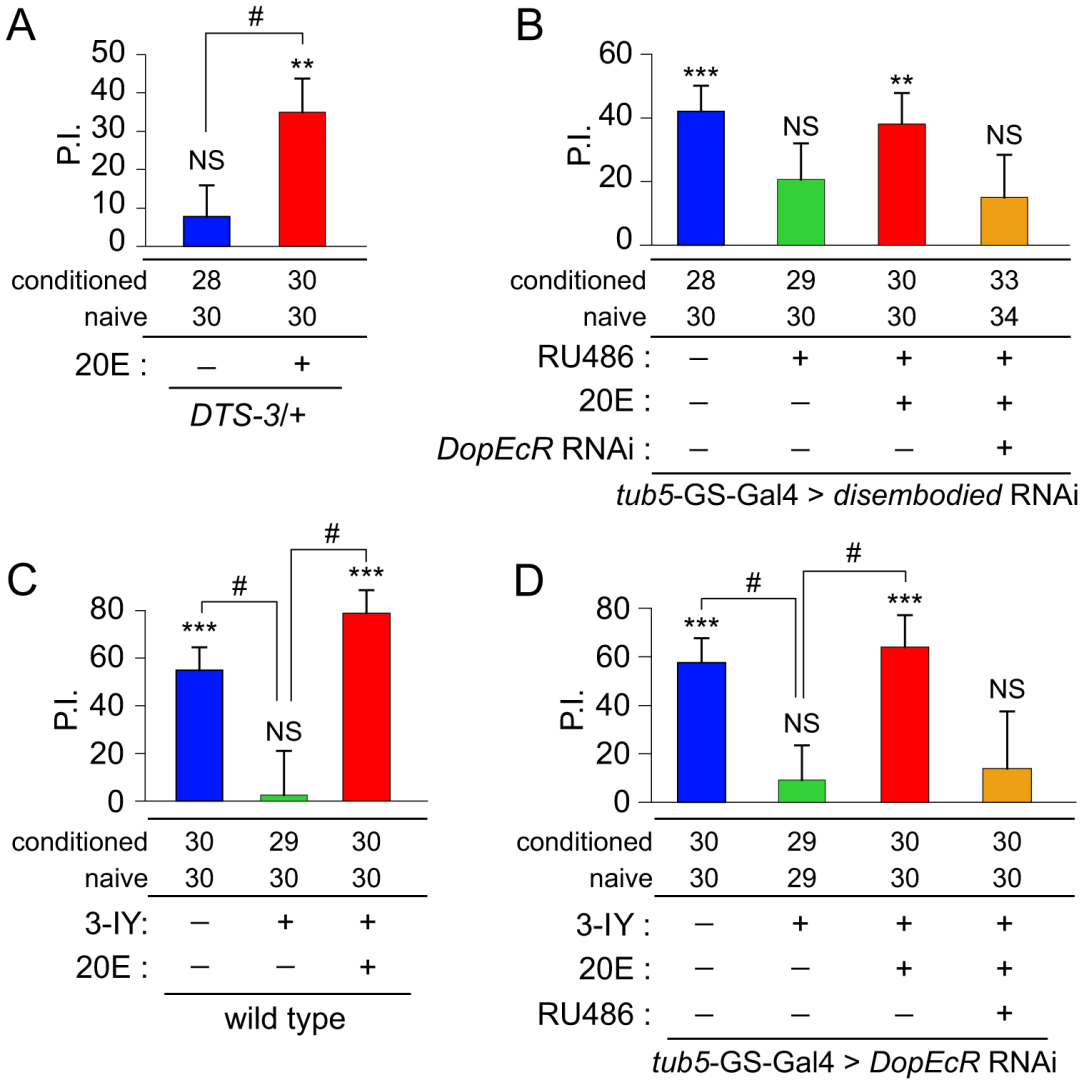
Impaired ecdysone synthesis causes a courtship memory defect. (A) Rescue of courtship memory in *DTS-3* males by feeding flies 20E prior to conditioning. (B) A defect in 30-minute courtship memory when the *dib* RNAi was expressed ubiquitously in adults, in response to RU486 application (*tub5*-GS-Gal4 driver). The *dib* RNAi-induced memory defect was not observed when flies were fed 20E, but was observed when 20E feeding was accompanied by the expression of the *DopEcR* RNAi. (C) Rescue of courtship memory in 3-iodo tyrosine (3-IY)-treated males by feeding flies 20E prior to conditioning. (D) Courtship memory in 3-IY-treated males was not restored by 20E when the *DopEcR* RNAi was expressed ubiquitously in adults. For 20E and RU486 feeding experiments, control flies received vehicle. The presence or absence of courtship memory was evaluated by applying the Mann–Whitney U-test to naïve and conditioned flies. Statistical significance is shown above each bar. NS, no significant difference. **, *P*<0.01; ***, *P*<0.001. Sample numbers for naïve and conditioned flies are shown under each graph. PIs were analyzed using Student's t-test or Krustal-Wallis One-Way ANOVA. #, *P*<0.05. Error bars (s.e.m.).

*Dominant temperature-sensitive 3* (*DTS-3*) is a dominant mutant allele of *molting defective* (*mld*; personal communication, P. Maroy, University of Szeged, Szeged, Hungary), a gene that encodes a putative transcription factor required for ecdysone biosynthesis [47], [48]. We previously reported that, unlike wild-type males, *DTS-3*/+ males did not exhibit an increase in 20E levels in response to 7-hour courtship conditioning, and that they were defective in long-term courtship memory (courtship LTM) [25]. As shown in Figure 5A, *DTS-3*/+ males did not show courtship suppression 30 minutes after 1-hour conditioning. Intriguingly, when *DTS-3*/+ males were fed 20E (0.1 mM) for 10 minutes immediately before courtship conditioning, the courtship-memory defect was rescued and courtship suppression was observed (*P* = 0.0043) (Figure 5A).

*disembodied* (*dib*) is one of the Halloween-family genes encoding the cytochrome P450 enzymes that are essential for ecdysone biosynthesis [49]. When *dib* expression was conditionally suppressed by treating mature adult males carrying the UAS-*dib* RNAi (gift from Dr. O'Connor, University of Minnesota) and *tub5*-GS-Gal4 with RU486, they exhibited a defect in courtship memory (Figure 5B). As with *DTS-3*/+ males, when *dib*-knockdown flies were fed 20E (0.1 mM) before courtship conditioning, they displayed experience-dependent courtship suppression (*P* = 0.006). In the *dib*-knockdown flies, this rescue effect of 20E was not observed when *DopEcR* expression was suppressed using the *DopEcR* RNAi (Figure 5B). Functional DopEcR is thus required for 20E-dependent courtship memory. These findings, together with the phenotypes of the *DopEcR*-mutant and *DTS-3*/+ males (Figure 5A), strongly suggest that ecdysone signaling plays a critical role in 30-minute courtship memory, and this signaling is mediated by DopEcR.

### DopEcR-mediated ecdysone signaling can compensate for the memory defect caused by dopamine deficiency

In addition to ecdysteroids, dopamine has been shown to be a direct ligand for DopEcR [29]. We fed flies 3-Iodotyrosine (3-IY) to block dopamine synthesis and examined the effect on courtship memory. As reported previously, courtship memory was defective in these flies [50], [51] (Figure 5C). We found that when the flies were additionally fed 20E (0.1 mM) 10 minutes before courtship conditioning, courtship memory was restored in spite of the block in dopamine synthesis (*P* = 0.00017; Figure 5C) and the PI was significantly increased (*P*<0.05; Figure 5C). The compensatory effect of 20E was also observed in 3-IY-treated flies of a different genetic background (Figure 5D). In contrast, when *DopEcR* RNAi was conditionally expressed in dopamine-depleted adults, 20E was not able to rescue courtship memory (Figure 5D). These results show that 20E compensates for the adverse effect of dopamine deficiency on courtship memory through the actions of DopEcR.

### Courtship memory defects in cAMP pathway mutants can be restored by modification of DopEcR-dependent ecdysone signaling

We next examined which intracellular signaling events are involved in the regulation of courtship memory by DopEcR. Here we focused our attention on the cAMP signaling pathway, because it plays a central role in learning and memory processes in diverse animal species [52]. We investigated whether 20E and DopEcR exert their effects on courtship memory via this signaling. The functional significance of cAMP for DopEcR-mediated signaling was indicated by a previous study in heterologous cell-culture systems, showing that DopEcR modulates intracellular cAMP levels in response to ligand binding [29]. One *Drosophila* gene that is crucial for regulating cAMP signaling is *rutabaga* (*rut*), which encodes a type I Ca^2+^/CaM-dependent adenylyl cyclase (AC) [53], [54]. Loss-of-function *rut* mutations result in lower cAMP-synthesizing activities and affect various forms of neural plasticity, including habituation of the GF pathway [36] and experience-dependent courtship suppression [55]. Habituation in the GF pathway was suppressed in both *DopEcR* and *rut* mutants (Figure 2C–E) [36], implying that the encoded proteins may have related functions in regulating neural plasticity.

**Figure 6:**
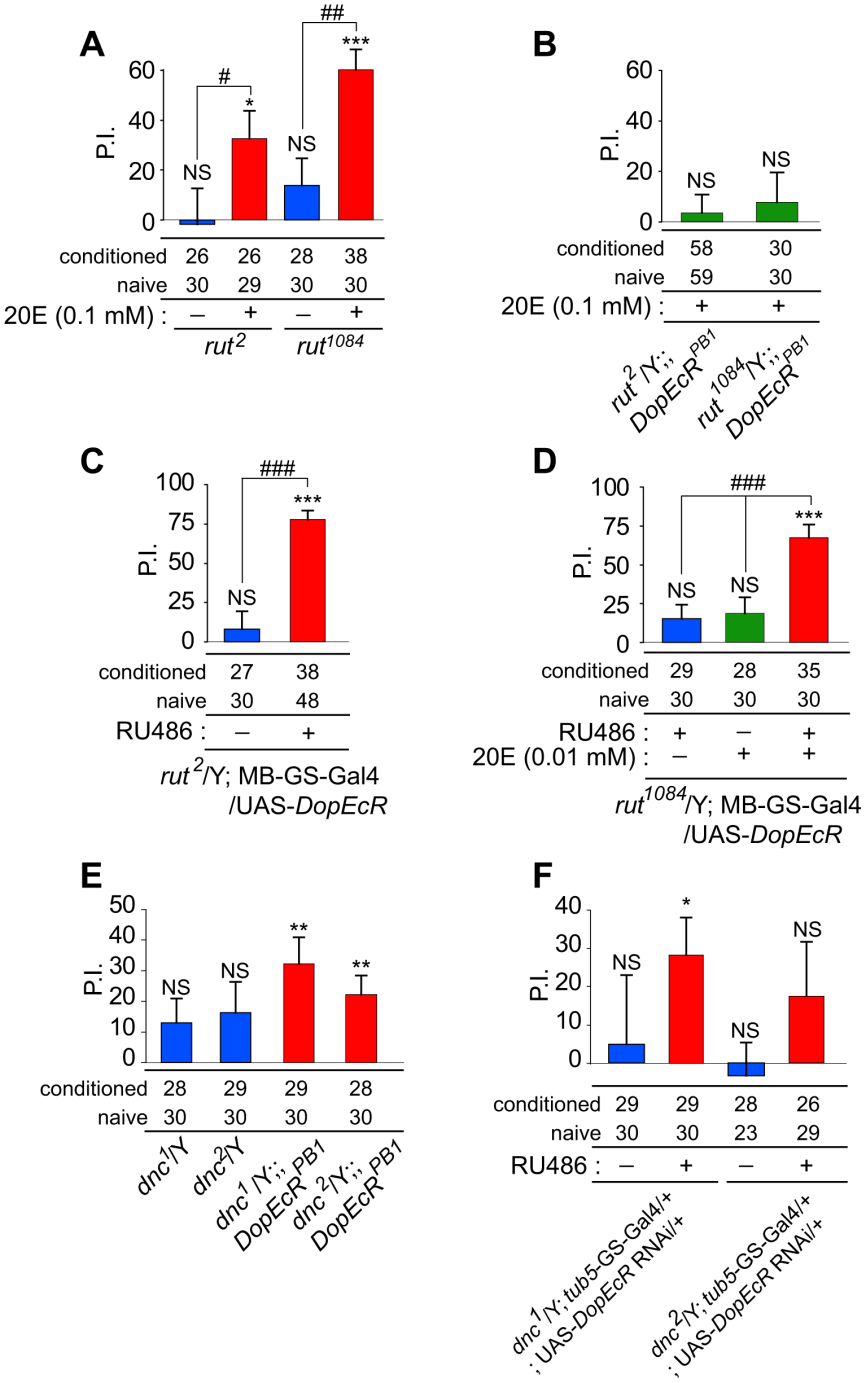
Effects of DopEcR-mediated signaling on courtship memory defects in cAMP-pathway mutants. (A) Rescue of defective courtship memory in flies with a hypomorphic *rut* allele (*rut^2^* or *rut^1084^*) by feeding them 20E prior to conditioning. (B) Introducing the *DopEcR^PB1^* mutation into the *rut* mutant backgrounds abolished the rescue effect of 20E on courtship memory. (C) The courtship memory defect in *rut^2^* was rescued by conditional overexpression of wild-type *DopEcR* in the adult MB neurons (MB-GS-Gal4 driver). (D) The courtship memory defect in *rut^1084^* was rescued by a combination of conditional *DopEcR* overexpression in the adult MB neurons and feeding the flies a lower dose (0.01 mM) of 20E. (E) Thirty-minute courtship memory was detected in *dnc* and *DopEcR^PB1^* double mutants. (F) Thirty-minute courtship memory was detected when the *DopEcR* RNAi was conditionally expressed in adult *dnc^1^* mutants using *tub*-GS-Gal4. For 20E and RU486 feeding experiments, control flies received vehicle. Courtship memory was evaluated by applying the Mann–Whitney U-test to naïve and trained flies. Statistical significance is shown above each bar. NS, no significant difference. *, *P*<0.05; **, *P*<0.01; ***, *P*<0.001. Sample numbers for naïve and conditioned flies are shown under each graph. PIs were analyzed using the Mann–Whitney U-test or Krustal-Wallis One-Way ANOVA. #, *P*<0.05; ##, *P*<0.01; ###, *P*<0.001 Error bars (s.e.m.).

Consistent with a previous report [42], males carrying a hypomorphic *rut* mutant allele (*rut^2^* or *rut^1084^*) were defective for courtship memory and showed no experience-dependent courtship suppression 30 minutes after 1-hour courtship conditioning (*P*>0.05; Figure 6A). Remarkably, the memory defect in *rut* mutants was restored when they were fed 20E (0.1 mM) for 10 minutes immediately before courtship conditioning (*P* = 0.0043 and 5.5×10^−5^ for *rut^2^* and *rut^1084^*, respectively; Figure 6A). The PIs for *rut^2^* and *rut^1084^* increased significantly following treatment with 20E (*P*<0.05 and *P*<0.01 for *rut^2^* and *rut^1084^*, respectively; Figure 6A). This pharmacological rescue of the *rut* memory phenotype was not observed in *rut* and *DopEcR^PB1^* double mutants (Figure 6B). These results strongly indicated that DopEcR mediates the compensatory effect of 20E on defective memory in *rut* mutants.

Considering the significance of the MB and *rut* for DopEcR-mediated memory processing, we examined their relationship. Courtship memory was analyzed in adult *rut^2^* mutants overexpressing *DopEcR* in the MB neurons. Courtship memory was restored by conditional overexpression of *DopEcR* using RU486-inducible MB-GS-GAL4 [56] (*P* = 1.4×10^−9^), and the PI increased significantly (*P*<0.001; Figure 6C). Although the memory defect in *rut^1084^*-mutant males was not rescued by solely overexpressing *DopEcR* in the MB (Figure 6D), feeding them a low concentration of 20E (0.01 mM) led to significant courtship suppression (*P* = 1.2×10^−6^; Figure 6D). Notably, administering 20E at this concentration was not sufficient to rescue the *rut^1084^* memory phenotype in the absence of *DopEcR* overexpression (Figure 6D, middle). The different requirements for rescuing courtship memory in *rut^2^* and *rut^1084^* may reflect differences in the severity of the mutations. Indeed, an olfactory-associated memory defect in *rut^1084^* mutants is similar to that in mutants of a presumptive *rut* null allele (*rut^1^*) [53], [57], whereas the *rut^2^* memory defect is milder [58], [59]. Overall, these findings demonstrate that the memory defect in *rut* mutants can be compensated by strengthening DopEcR-mediated ecdysone signaling in MB neurons.

Another “memory gene” involved in cAMP signaling is *dunce* (*dnc*), which encodes a cAMP-specific phosphodiesterase (PDE) that is required for cAMP degradation [60], [61]. Like *rut* mutants, *dnc* loss-of-function mutants are defective for various types of neuronal and behavioral plasticity [36], [61], [62]. In contrast to *rut* mutants, *dnc* mutants display an increased rate of GF habituation [36], possibly reflecting the fact that *rut* and *dnc* mutations have opposite effects on cAMP levels.

As shown previously [42], hypomorphic *dnc* mutants (*dnc^1^* and *dnc^2^*) did not exhibit experience-dependent courtship suppression 30 minutes after 1-hour courtship conditioning, and were therefore defective for courtship memory (Figure 6E). Notably, double mutants carrying both *dnc* and *DopEcR* loss-of-function mutations displayed courtship suppression (*P* = 0.0016 for *dnc^1^*/Y; *DopEcR^PB1^* and 0.002 for *dnc^2^*/Y; *DopEcR^PB1^*; Figure 6E). In addition, *dnc^1^* males displayed courtship memory, which manifests as significant experience-dependent courtship suppression (*P* = 0.0274) when *DopEcR* was conditionally down-regulated using *tub5*-GS-Gal4 in conjunction with the *DopEcR* RNAi (Figure 6F). *dnc^2^* males showed a similar tendency when *DopEcR* was down-regulated, although the difference in CIs between naïve and conditioned flies was not statistically significant. Overall, these experimental results with *rut* and *dnc* mutants strongly suggest that DopEcR exerts its critical function in courtship memory by regulating cAMP signaling pathway.

### cAMP levels in the mushroom body rapidly increase in a DopEcR-dependent manner after 20E feeding

**Figure 7:**
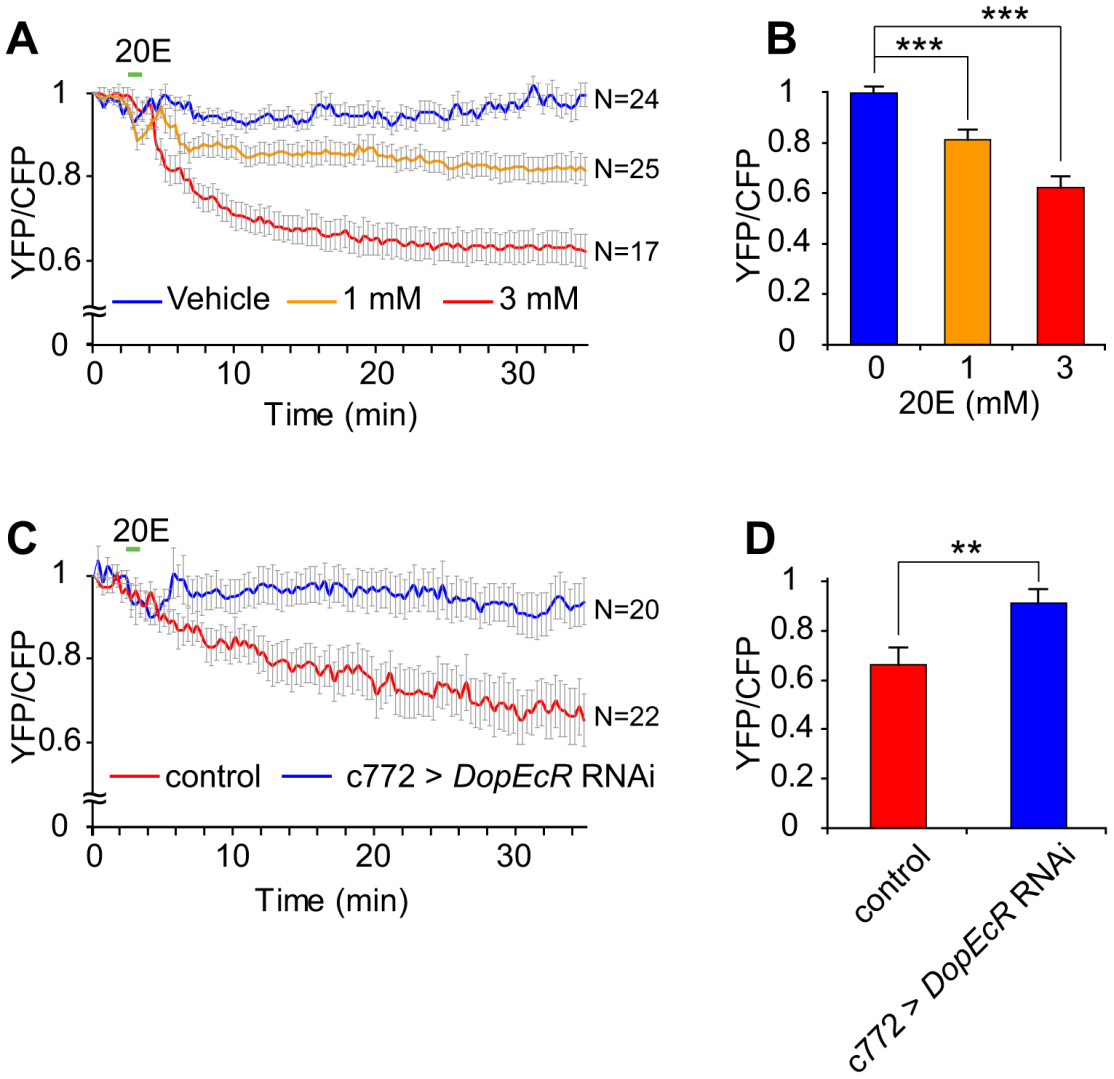
20E-feeding triggers elevated cAMP levels in MB neurons. (A) 20E-induced increase in cAMP levels in MB neurons, as monitored by the cAMP bio-indicator Epac1-camps. Time courses of averaged YFP/CFP ratios in response to vehicle control (blue, N = 24), 1 mM 20E (orange, N = 25) and 3 mM 20E (red, N = 17) are shown, with s.e.m. (B) Averaged YFP/CFP ratios 30 minutes after 20E or vehicle feeding. One-Way ANOVA followed by post-hoc comparisons. ***; *P*<0.001. (C, D) Expression of the *DopEcR* RNAi suppressed the 20E-induced increase in cAMP levels in MB neurons. Time courses of averaged YFP/CFP ratios (C), and the ratios 30 minutes after 20E feeding (D), for control (red, N = 20) and MB-specific *DopEcR* knockdown (blue, N = 22) lines. Student's t-test. **; *P*<0.01. Error bars (s.e.m).

The courtship-memory defect in *rut* mutants can be attributed to their inability to appropriately increase intracellular cAMP levels during courtship conditioning. Because 20E feeding and *DopEcR* overexpression resulted in restoration of courtship memory in *rut* mutants, we hypothesized that strengthening DopEcR-mediated ecdysone signaling would increase in cAMP levels in brain regions critical for memory processing, such as the MBs. To investigate this possibility, we examined the effects of 20E feeding on cAMP levels in the MBs of live adult flies using UAS-Epac1-camps, a Förster (fluorescence) resonance energy transfer (FRET)-based cAMP reporter [63]. The reporter was expressed in MBs using c772, one of the MB drivers that were effective in the rescue and phenocopy experiments described above (Figure 4). Immediately after the flies were fed 20E, the cAMP levels in the MBs were assessed by cAMP-induced changes in FRET, as the ratio between YFP and CFP signals. In wild-type males, feeding either 3 mM or 1 mM 20E caused a time- and dose-dependent decrease in the average FRET (Figure 7A). The effects of 20E on FRET are statistically significant (*P*<0.001; Figure 7B). Because a decrease in FRET corresponds to an increase in cAMP levels, our results indicated that 20E increases cAMP levels in the MB. This effect was eliminated by simultaneously expressing the *DopEcR* RNAi in the MB (Figure 7C and 7D). These data indicate that the activation of ecdysone signaling rapidly increases cAMP levels in the MBs, and that it does so through DopEcR.

## Discussion

Here we used genetic, pharmacological, and behavioral approaches in *Drosophila* to demonstrate that the steroid hormone 20E rapidly regulates behavioral plasticity via a non-genomic mechanism that is mediated by the GPCR-family protein DopEcR. This non-canonical steroid signaling pathway was found to have strong functional interactions with the classical “memory genes” *rut* and *dnc*, which encode the central components of the cAMP pathway. The identification of 20E as an important modulator of cAMP signaling in the adult *Drosophila* brain reveals an unprecedented opportunity—that of taking advantage of fly genetics to dissect the molecular and cellular mechanisms responsible for the non-genomic steroid signaling that underlies neuronal and behavioral plasticity.

### Physiological effects of DopEcR-mediated signaling

Our electrophysiological analyses revealed that the GF pathway of *DopEcR* mutant flies is more resistant to habituation than that of control flies (Figure 2). Direct excitation of GF or its downstream elements would lead to a short-latency response of the DLM, which could follow high-frequency stimuli up to several hundred Hz [32], [33], [36]. In contrast, the afferent input to the GF leads to a long-latency response that is labile and fails to follow repetitive stimulation well below 100 Hz and displays habituation even at 2–5 Hz [36]–[40]. Although there is the possibility that DopEcR-positive thoracic neurons may modulate thoracic motor outputs and contribute to certain parameters of the habituation process not characterized in this study, the more effective modulation would occur in the more labile element afferent to the GF circuit rather than the robust GF-PSI-DLMn downstream pathway, which is responsible for the reliability of the escape reflex. Thus, the mutant phenotype in habituation indicates that DopEcR positively controls activity-dependent suppression of neuronal circuits afferent to the GF neurons in the brain.

Moreover, our finding that *DopEcR* and *rut* mutants have a similar GF habituation phenotype raises the possibility that DopEcR positively regulates cAMP levels in the relevant neurons following repetitive brain stimulation. Besides GF habituation, *Drosophila* displays olfactory habituation, which is mediated by the neural circuit in the antennal lobe [38]. Interestingly, Das et al. found that olfactory habituation is induced by enhancement of inhibitory GABAergic transmission, and that *rut* function is required for this neuronal modulation [64]. Similar modulation of GABAergic transmission may also be responsible for habituation of the GF pathway. It will be interesting to examine whether and how DopEcR contributes to the regulation of *rut* and enhanced GABAergic transmission in GF habituation.

Several studies already suggested that 20E has rapid, EcR-independent effects in *Drosophila* and other invertebrate species. For example, 20E was shown to reduce the amplitude of excitatory junction potentials at the dissected *Drosophila* larval neuromuscular junction (NMJ), and to do so within minutes of direct application [65]. Whereas treatment with 20E did not change the size and shape of the synaptic currents generated by spontaneous release, it led to a reduction in the number of synaptic vesicles released by the motor nerve terminals following electrical stimulation [65]. A similar effect of 20E was observed in crayfish, and it was suggested that the suppression of synaptic transmission by 20E may account for the quiescent behavior of molting insects and crustaceans [66]. These observations suggested that 20E suppresses synaptic efficacy under certain conditions by modulating presynaptic physiology through a non-genomic mechanism. It is possible that such actions of 20E are mediated by DopEcR. To detail the mechanisms underlying DopEcR-dependent neural plasticity, it will be worthwhile to determine if and how DopEcR contributes to 20E-induced, rapid synaptic suppression at the physiologically accessible larval NMJ, and to determine the extent to which non-genomic mechanisms of steroid actions are shared between the larval NMJ and the adult brain.

### Functional interactions between DopEcR and the cAMP pathway

One surprising finding made in this study is that ecdysone signaling can modify the phenotypes associated with mutations in the classic “memory genes”, namely *rut* and *dnc*, through the actions of DopEcR. *rut* and *dnc* encode central components of the cAMP pathway, which is required for memory processing in vertebrates as well as invertebrates. Our demonstration that genetically and/or pharmacologically enhancing DopEcR-mediated ecdysone signaling restores the courtship memory phenotype of loss-of-function *rut* mutants (Figure 6A–D) suggests that 20E-mediated DopEcR activation triggers an outcome similar to *rut* activation, i.e., increased cAMP levels. This assumption is supported by our finding that loss-of-function *dnc* mutants restore courtship memory when DopEcR activity is suppressed (Figure 6E and 6F). A similar restoration of the *dnc* memory phenotype was previously reported in a *dnc* and *rut* double mutant [58], again supporting the idea that DopEcR positively regulates cAMP production.

The results of rescue and phenocopy experiments (Figure 4) indicate that the MB is critical for the DopEcR-dependent processing of courtship memory. Although the endogenous expression pattern of DopEcR is not known, DopEcR is thus likely to modulate cAMP levels in the MB in response to 20E during courtship conditioning. We have recently generated a new Gal4 line, in which a portion of the first coding exon of DopEcR is replaced with a DNA element that contains the Gal4 cDNA whose translation initiation codon is positioned exactly at the DopEcR translation start site (Q. Li and Y. Rao are preparing a paper describing the details of this Gal4 line). When this line was used to drive UAS-GFP, the reporter gene was widely expressed in the adult brain with prominent signals in the MB (unpublished observation). This preliminary result strongly indicates the endogenous expression of DopEcR in the MB. We have also directly shown that cAMP levels in the MB increase rapidly in flies fed 20E (Figure 7A), and that this increase does not occur when DopEcR expression is down-regulated specifically in the MB (Figure 7B). Taken together, these findings suggest that DopEcR expressed in the MB responds to 20E and acts upstream of cAMP signaling in a cell-autonomous manner.

Surprisingly, enhancement of DopEcR-mediated ecdysone signaling restored courtship memory in flies harboring a strong hypomorphic allele of *rut* (*rut^1084^*) (Figure 6A and 6D). A similar result was obtained even in mutants harboring a presumptive *rut* null allele *rut^1^* (data not shown). These results suggest that, upon stimulation by 20E, DopEcR may be able to signal via another adenylyl cyclase that can compensate for the lack of Rut. This interesting possibility requires further investigation.

### Dopamine and DopEcR-mediated ecdysone signaling in the regulation of courtship memory

In this study, we have focused on the roles and mechanisms of action of DopEcR-mediated, non-genomic ecdysone signaling. As we previously found that 20E levels rise in the head during courtship conditioning [25], the data presented here suggest that DopEcR is activated by 20E during conditioning, triggers a rise in cAMP levels and induces physiological changes that subsequently suppress courtship behavior. This interpretation assumes that 20E directly activates DopEcR to increase cAMP levels. Previous cell-culture studies suggested that DopEcR also responds to dopamine to modulate intracellular signaling [29]. Furthermore, Inagaki et al. have demonstrated that flies respond to starvation by sensitizing gustatory receptor neurons to sugar via dopamine/DopEcR signaling [30]. We thus need to consider whether dopamine is directly involved in the processing of courtship memory through DopEcR. There is a possibility that 20E initially stimulates the production and/or release of dopamine, and that it in turn activates DopEcR and elevates cAMP levels to induce courtship memory. We think that this possibility is unlikely because even when courtship memory is disrupted by pharmacological suppression of dopamine synthesis, 20E feeding can compensate for decreased dopamine and allow restoration of memory (Figure 5C and 5D). Although dopamine plays a significant role in courtship memory [50], our results suggest that DopEcR does not act as the major dopamine receptor in this particular learning paradigm. We thus favor the possibility that dopamine contributes to courtship memory in parallel with, or upstream of, DopEcR-mediated ecdysone signaling. Consistent with this view, Keleman et al. reported that the formation of courtship memory depends on the MB γ neurons, which express DopR1 dopamine receptors, receiving dopaminergic inputs [51]. Notably, our results indicate that the processing of courtship memory requires DopEcR expression in the αβ, but not γ, neurons of the MB (Figure 4), which makes it unlikely that DopEcR is directly influenced by the dopaminergic neurons innervating γ neurons.

### Ecdysone signaling mediated by DopEcR and EcR

Ecdysone signaling through nuclear EcRs is necessary for forming long-term courtship memory that lasts at least 5 days, but appears not to have a significant effect on short-term courtship memory [25]. In contrast, we found that DopEcR-mediated ecdysone signaling is critical for habituation and 30-minute courtship memory. These findings suggest that DopEcR and EcRs control distinct physiological responses to courtship conditioning, and that the former regulates short-term memory, while the latter regulates long-term memory. Although non-genomic actions of steroid hormones have been implicated in vertebrate learning and memory [67], [68], such actions have been attributed mainly to the classical nuclear hormone receptors that function outside of the nucleus and exert roles distinct from those of steroid-activated transcription factors [12]. Although recent evidence has shown that membrane-bound receptors independent of the classical estrogen receptors are involved in estradiol-induced consolidation of hippocampal memory [69], the molecular identities of these proteins have not been established. Our findings here provide a novel framework for dissecting GPCR-mediated steroid signaling at the molecular and cellular levels. Furthermore, future analysis of the functional interplay between genomic and non-genomic steroid signaling pathways is expected to reveal novel mechanisms through which steroid hormones regulate plasticity of the nervous system and other biological phenomena.

## Materials and Methods

### Fly stocks

Flies were reared at 25°C and 64% humidity, in a 12-hour light/dark cycle and on a conventional glucose-yeast-cornmeal agar medium. The *DopEcR^PB1^* strain used in this study was produced by outcrossing with Cantonized *w* mutant flies. The *DopEcR*-Gal4 and UAS-*DopEcR* strains were generated in this study. For *DopEcR*-Gal4, the putative promoter region of *DopEcR* (a 588-bp upstream sequence) was fused to the yeast *Gal4* gene. For UAS-*DopEcR*, the *DopEcR* coding sequence was inserted downstream of the UAS (upstream activating sequence) in the pUAST vector. Other fly strains used in this study were obtained from the following sources: c772, c739, 201 y, UAS-CD4-tdGFP and UAS-Epac1-camps (55A) (Bloomington *Drosophila* Stock Center); *tub5*-GS-Gal4 (Scott D. Pletcher, Baylor College of Medicine, Houston, TX, USA); MB-GS-Gal4 (Ronald L. Davis, The Scripps Research Institute, Jupiter, FL, USA); UAS-*DopEcR* RNAi (VDRC); UAS-*disembodied* RNAi (Michael B. O'Connor University of Minnesota, Minneapolis, MN, USA); *DTS-3* and Samarkand (Anne F. Simon, Western Ontario University, Ontario, Canada). The Canton-S (2202u) strain was used as the wild-type control.

### Immunohistochemical analysis

Adult brains were dissected from 3 to 5-day-old male flies in PBS and fixed for 1 hour with 3.7% formaldehyde at 25°C, in PBS containing 0.05% Triton X-100 (PBST). The brains were blocked with PBST containing 0.1% normal goat serum for 1 hour. Rabbit anti-GFP antibody (1∶1000; A11122, Invitrogen) was used for the primary antibody. The brains were counter-stained with nc82, the mouse anti-Bruchpilot antibody (1∶20; Developmental Studies Hybridoma Bank, University of Iowa). Alexa Fluor 555-conjugated anti-rabbit IgG (1∶300; Invitrogen) and Alexa 647-conjugated anti-mouse IgG (1∶300; Invitrogen) were used as secondary antibodies for detection of anti-GFP and nc82, respectively. Images were acquired as a z-stack, using FV1000 confocal microscope (Olympus). Volume-rendered images were displayed using FluoRender (http://www.fluorender.com).

### RT-PCR

Total RNA was prepared from 20 fly heads of each genotype using TRIzol solution (Invitrogen), and subjected to a reverse transcription reaction using a poly-dT_20_ primer and Superscript II enzyme (Invitrogen), according to the manufacturer's instructions. The *DopEcR* cDNA sequence was amplified by PCR using the following primers: forward, 5′-ATGCAGGAAATGAGCTACCT-3′ and reverse, 5′-CTAGTCATCTGGGTCCAACC-3′. *rp49* was used as the internal control (forward, 5′-ATGACCATCCGCCCAGCA-3′ and reverse, 5′-AATCTCCTTGCGCTTCTTGG-3′). The gel images were processed using ImageJ software, to estimate the quantity of PCR products.

### Electrophysiology

The preparation of flies, stimulation, recording, and analysis of muscle responses were performed as described previously [36], with some modifications. Electrical stimuli (0.1 msecond pulse) were delivered across the brain through two uninsulated tungsten electrodes inserted in the eyes (anode normally in the right eye). The action potentials in the left side leg extensor (TTM) and the right side wing depressor (DLM) were recorded as an indicator of GF pathway output [36]. Flies were given tissue paper balls (less than 1 mm in diameter) to inhibit flight, but were free to perform normal jump-and-escape reflexes. All recordings were carried out in an experimental Faraday cage covered with a black plastic sheet to reduce ambient light. To minimize the possible effects of handling and anesthesia, flies mounted for recording were rested for at least 1 hour in a humid chamber before recording. After being assessed for response thresholds (during an inter-stimulus interval, ISI, of 30 seconds), flies were rested for 5 minutes before the habituation test. Three classes of responses, with progressively greater thresholds, were identified: long-latency, intermediate-latency, and short-latency. These responses could easily be distinguished in individual flies, and were used as an “internal gauge” on which to base the stimulation intensity for the habituation test. For each test, the stimulus intensity was set at the mean value of the thresholds for the long-latency and short-latency. To avoid causing artifacts by improper handling of flies, flies that had abnormally high activities or failed to respond more than twice, consecutively, were excluded from data analysis. Dishabituation stimuli (air puffs) were provided by gently squeezing a rubber bulb connected, by tubing, to a pipette nozzle mounted 2 cm to the anterior-left of the fly. All habituation data were recorded using the software pCLAMP 5, and analyzed with clampfit in pCLAMP10. The cumulative curves of the habituation responses were plotted with custom-designed software on the Matlab 7 platform.

### Courtship-conditioning assay

The courtship conditioning assay was performed at 25°C and 64% humidity, in an environment room under white light, as described previously [25], with some modifications. All males were 3–5 days old at the time of testing. They were anesthetized with CO_2_ and stored in isolation for at least 24 hours prior to experiments. Females used as “trainers” in courtship conditioning were 3-days old and were fertilized a day before conditioning. In the conditioning phase, virgin males were placed with unreceptive, non-virgin females (or alone in ‘pseudo-training’ experiments for naïve control males), in single-pair-mating chambers containing food medium (15 mm diameter×5 mm in depth), for 1 hour. After conditioning, males were rested individually for 30 minutes, in a glass tube (12 mm in diameter ×75 mm in depth, VWR International) containing food medium. Memory tests were performed in a courtship chamber (15 mm in diameter ×3 mm in depth) containing a freeze-killed virgin female. The male courtship behaviors were videotaped for a 10-minute test period, using DVD camcorder (Sony DCR-DVD105), and were manually scored for courtship index (CI). The CI was defined as the proportion of time spent for courtship behaviors (orientation, tapping, singing, licking and copulation attempts). We did not exclude from analysis males with a low courtship level. To compare CIs for conditioned and naïve males, we analyzed the data non-parametrically, using the Mann-Whitney U test, because the CI values were often not distributed normally. When CIs for conditioned and naïve males were significantly different (*P*<0.05), male courtship behavior was considered to be suppressed in an experience-dependent manner (courtship memory). Experimental data are presented in the figures as the performance index (PI), which was calculated using the following formula (after CIs were subjected to arcsine square root transformation to approximate normal distributions): PI = 100×(CI^Ave naïve^−CI^conditioned^)/CI^Ave naïve^, where CI^Ave naïve^ and CI^conditioned^ represent the averaged CI for naïve flies and a CI for each conditioned fly, respectively. Naïve courtship levels of Canton-S, *DopEcR^PB1^* and *DopEcR* RNAi (UAS-*DopEcR* RNAi/+; *tub5*-GS-Gal4/+) flies were shown in Table S1. The CIs were not statistically different between Canton-S and *DopEcR^PB1^* (*P* = 0.086) and there was no statistical difference between the CIs of *DopEcR* RNAi males with or without RU486 treatment (*P* = 0.8459). Mann-Whitney U test.

### Drug treatment

Flies carrying the RU486-inducible transgene (GeneSwitch strains) were fed food containing 500 µM RU486 (Mifepristone, Sigma) or vehicle (ethanol; final concentration <2%) for 3 days prior to the experiment. 20E was fed for 10 min using Kimwipe paper soaked in 1M sucrose solution containing a particular concentration of 20E (Sigma). The 20E stock solution (25 mM) was prepared in ethanol. 3-Iodotyrosine (3-IY) was mixed into yeast paste with a final concentration of 10 mg/ml. Up to 10 newly eclosed flies were placed in vials containing fly food with 3-IY-yeast paste for 4 days.

### Live cAMP imaging

The change in cAMP levels was monitored using the genetically encoded cAMP reporter Epac1-camps [63]. This reporter was expressed in MB neurons using the c772-GAL4 driver. Two α-lobe tips and clusters of calix cell bodies were set as a region of interest (ROI), and observed through the head cuticle. Test flies were immobilized on an observation plate by gluing the dorsal portion of the head and neck with nail polish. The observation plate was a large glass coverslip (24×60 mm) attached to a small plastic coverslip (22×22 mm) with a hole (7 mm diameter). The fly thorax was positioned at the edge of the hole so that the fly head was directly attached to the glass coverslip. Confocal images were obtained using a Plan-Neofluar 20× objective on a Zeiss 510 inverted confocal microscope (Zeiss, Oberkochen, Germany). Epac1-camps fluorescence was scanned with a 458 nm Argon ion laser line. YFP-FRET and CFP-donor emissions were separated by means of a NFT545 dichroic mirror and BP475-525 and LP560 emission filters. YFP and CFP signals were scanned simultaneously onto separate photomultiplier tubes, and obtained every 20 seconds. After 3 minutes of baseline FRET (YFP to CFP ratio) measurement, the test fly was fed 20E-sucrose solution or vehicle control for 1 minute using a Kimwipe (10 mm×10 mm) soaked with the solution. The 20E-sucrose solution contained blue food dye (Acid Blue 9, 0.125 mg/ml) as an indicator of ingestion. The effects of 20E on FRET were observed for 30 minutes and analyzed as described by Shafer et al [63]. To compare the FRET time-course among different experiments, the YFP/CFP ratio values were normalized to the value of the first time-point.

## Supporting Information

Figure S1 The motor ability of *DopEcR^PB1^* flies is normal. The climbing assay was performed in the dark as described previously using a counter-current apparatus [S1]. Twenty flies were placed in the plastic “start” vial, and gently tapped to the bottom. The apparatus was laid in a horizontal position and the flies were permitted to climb toward a distal vial for 30 seconds. Afterwards, the tubes were misaligned, trapping the flies in either the start vial or the 2^nd^ vial. This procedure was repeated five times, separating the flies into six vials. The number of flies in each vial was counted. Flies that climbed to all five distal vials received a score of five, whereas flies remaining in the start vial receive a score of zero. The climbing index was calculated as the average of scores from all flies tested. The Mann–Whitney U-test was applied for statistical analysis.
(DOCX)

Figure S2 *DopEcR* transcript levels are effectively suppressed by RNA interference. The efficacy of the *DopEcR* RNAi was evaluated by RT-PCR. The expression of the dsRNA targeting *DopEcR* transcripts was controlled by applying RU486 to activate the *tub5*-GS-Gal4 driver. *rp49* served as an internal control. The level of *DopEcR* transcripts was significantly decreased by RU486 application.
(PDF)

Table S1 Naïve courtship indices of Canton-S, *DopEcR^PB1^* and *DopEcR* RNAi (UAS-*DopEcR* RNAi/+; *tub5*-GS-Gal4/+) males.
(DOCX)
