# Supplementary material for: A Novel Role for Ecdysone in Drosophila Conditioned Behavior: Linking GPCR-Mediated Non-canonical Steroid Action to cAMP Signaling in the Adult Brain
Source: PLoS Genet. 2013 Oct 10;9(10):e1003843. doi: 10.1371/journal.pgen.1003843 (PMC3794910; doi:10.1371/journal.pgen.1003843)
Supplement: Figure S1 — The motor ability of DopEcRPB1 flies is normal. The climbing assay was performed in the dark as described previously using a counter-current apparatus [S1]. Twenty flies were placed in the plastic “start” vial, and gently tapped to the bottom. The apparatus was laid in a horizontal position and the flies were permitted to climb toward a distal vial for 30 seconds. Afterwards, the tubes were misaligned, trapping the flies in either the start vial or the 2nd vial. This procedure was repeated five times, separating the flies into six vials. The number of flies in each vial was counted. Flies that climbed to all five distal vials received a score of five, whereas flies remaining in the start vial receive a score of zero. The climbing index was calculated as the average of scores from all flies tested. The Mann–Whitney U-test was applied for statistical analysis. (DOCX) [file pgen.1003843.s001.docx]

S1. Boynton S, Tully T (1992) latheo, a new gene involved in associative learning and memory in Drosophila melanogaster, identified from P element mutagenesis. Genetics 131: 655-672.
