# Supplementary figures and images for: A Novel Role for Ecdysone in Drosophila Conditioned Behavior: Linking GPCR-Mediated Non-canonical Steroid Action to cAMP Signaling in the Adult Brain
Source: PLoS Genet. 2013 Oct 10;9(10):e1003843. doi: 10.1371/journal.pgen.1003843 (PMC3794910; doi:10.1371/journal.pgen.1003843)

UAS-*DopEcR* RNAi;  
*tub5*-GS-Gal4

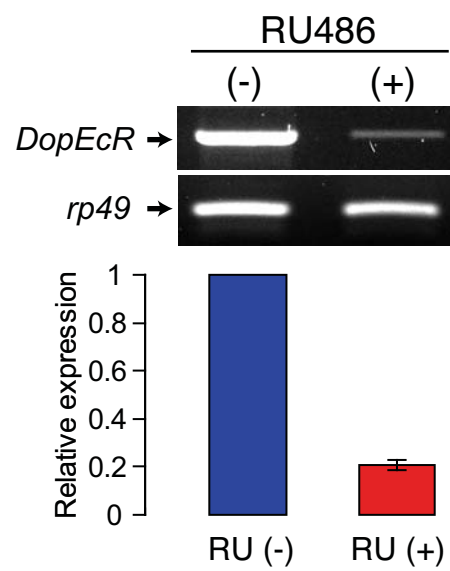

Supplement: Figure S2 — DopEcR transcript levels are effectively suppressed by RNA interference. The efficacy of the DopEcR RNAi was evaluated by RT-PCR. The expression of the dsRNA targeting DopEcR transcripts was controlled by applying RU486 to activate the tub5-GS-Gal4 driver. rp49 served as an internal control. The level of DopEcR transcripts was significantly decreased by RU486 application. (PDF) [file pgen.1003843.s002.pdf]
