# Supplementary material for: A Novel Role for Ecdysone in Drosophila Conditioned Behavior: Linking GPCR-Mediated Non-canonical Steroid Action to cAMP Signaling in the Adult Brain
Source: PLoS Genet. 2013 Oct 10;9(10):e1003843. doi: 10.1371/journal.pgen.1003843 (PMC3794910; doi:10.1371/journal.pgen.1003843)
Supplement: Table S1 — Naïve courtship indices of Canton-S, DopEcRPB1 and DopEcR RNAi (UAS-DopEcR RNAi/+; tub5-GS-Gal4/+) males. (DOCX) [file pgen.1003843.s003.docx]

| Genotype | Courtship Index | | | | | |
| --- | --- | --- | --- | --- | --- | --- |
|  | Maximum | 75% | Median | 25% | Minimum | N |
| Canton-S | 0.993 | 0.955 | 0.860 | 0.570 | 0 | 59 |
| *DopEcR^PB1^* | 0.992 | 0.906 | 0.798 | 0.583 | 0 | 30 |
| UAS-*DopEcR* RNAi/+; *tub5*-GS-Gal4/+ (RU-) | 0.968 | 0.866 | 0.624 | 0.020 | 0 | 30 |
| UAS-*DopEcR* RNAi/+; *tub5*-GS-Gal4/+ (RU+) | 0.985 | 0.811 | 0.625 | 0.019 | 0 | 28 |
